# Disentangling the associations between parental BMI and offspring body composition using the four‐component model

**DOI:** 10.1002/ajhb.22825

**Published:** 2016-02-05

**Authors:** Delan Devakumar, Carlos Grijalva‐Eternod, Mario Cortina‐Borja, Jane Williams, Mary Fewtrell, Jonathan Wells

**Affiliations:** ^1^ UCL Institute for Global HealthLondonWC1N 1EHUnited Kingdom; ^2^ Clinical Epidemiology, Nutrition and Biostatistics SectionUCL Institute of Child HealthLondonWC1N 1EHUnited Kingdom; ^3^ Childhood Nutrition Research Centre, UCL Institute of Child HealthLondonWC1N 1EHUnited Kingdom

## Abstract

**Objectives:**

This study sets out to investigate the intergenerational associations between the body mass index (BMI) of parents and the body composition of their offspring.

**Methods:**

The cross‐sectional data were analyzed for 511 parent–offspring trios from London and south‐east England. The offspring were aged 5–21 years. Parental BMI was obtained by recall and offspring fat mass and lean mass were obtained using the four‐component model. Multivariable regression analysis, with multiple imputation for missing paternal values was used. Sensitivity analyses for levels of non‐paternity were conducted.

**Results:**

A positive association was seen between parental BMI and offspring BMI, fat mass index (FMI), and lean mass index (LMI). The mother's BMI was positively associated with the BMI, FMI, and LMI *z*‐scores of both daughters and sons and of a similar magnitude for both sexes. The father's BMI showed similar associations to the mother's BMI, with his son's BMI, FMI, and LMI *z*‐scores, but no association with his daughter. Sensitivity tests for non‐paternity showed that maternal coefficients remained greater than paternal coefficients throughout but there was no statistical difference at greater levels of non‐paternity.

**Conclusions:**

We found variable associations between parental BMI and offspring body composition. Associations were generally stronger for maternal than paternal BMI, and paternal associations appeared to differ between sons and daughters. In this cohort, the mother's BMI was statistically significantly associated with her child's body composition but the father's BMI was only associated with the body composition of his sons. Am. J. Hum. Biol. 28:524–533, 2016. © 2016 The Authors American Journal of Human Biology Published by Wiley Periodicals, Inc.

The association between the body mass index (BMI) of parents and offspring has been of interest in relation to the worldwide rising trend of obesity. It is generally understood that BMI tracks from one generation to the next (Reilly et al., 2005) and that parental obesity strongly predicts offspring childhood obesity (Parsons et al., 1999).

However, several issues remain poorly understood. There is little information on what underlies such intergenerational correlations in BMI at the level of specific tissues, such as lean mass (LM) and fat mass (FM). While BMI is a useful indicator at a population level, it is a poor predictor of adiposity, as it incorporates both FM and LM but cannot differentiate their ratio (Wells, 2000; Wells et al., 2006). Thus, parent‐offspring correlations might reflect consistency in adiposity, or in frame size and muscularity. The evidence is mixed as to whether a sex‐ or gender‐specific association between parent and child exists, as discussed in more detail below, and a number of different mechanisms may be involved, with both environmental (Power et al., 2011) and genetic factors (Schousboe et al., 2003) transmitting parental effects to the child's phenotype. Indeed, each parent might influence its offspring through different behavioral and biologically mediated mechanisms, with boys and girls responding to different cues.

The possibility that parental BMI may show different associations with the phenotype of sons and daughters can be disentangled into two discrete components, and again a number of different mechanisms may be relevant. First, paternal and maternal BMI might show contrasting associations with child phenotype. The genetic basis of obesity is assumed to derive from the accumulation of risk alleles, where each risk allele contributes a relatively small effect to BMI (Elks et al., 2012). In general, Mendelian inheritance is not sex specific; however, genetic imprinting is one pathway by which parents can exert different influences on their offspring. In this case, the allele from one parent is active, while the other is switched off. The placenta is thought to be particularly important for the control of imprinting, either as a temporary or permanent phenomenon (Frost and Moore, 2010). Programming effects may also occur. Stronger associations between maternal than paternal BMI and offspring BMI, reported in some but not all studies (Danielzik et al., 2002; Lawlor et al., 2007; Linabery et al., 2013; Whitaker et al., 2010), may indicate the influence of maternal physiology during pregnancy or lactation. However, imprinting of the sperm is also possible, and exposures such as paternal famine or smoking during adolescence have been associated with offspring phenotype (Northstone et al., 2014; Pembrey et al., 2006). Finally, the two parents might generate contrasting behavioral influences on offspring lifestyle. For instance, maternal attitudes, such as disinhibition, are known to influence child eating behaviors (Cutting et al., 1999).

Second, sons and daughters might respond in contrasting ways to parental phenotype. Even during early childhood, boys within the normal range of nutritional status tend to have greater fat‐free mass than girls (Wells et al., 2012), while the tissue composition of excess weight gain also differs between the sexes (Wells et al., 2006). Such differences may relate to sex‐different sensitivity to hormones such as insulin and leptin (Wells and Cole, 2014). In terms of developmental mechanisms, differential in utero DNA methylation of the fetus has been shown between the sexes (Cooper et al., 2012; Khulan et al., 2012). Evidence showing that paternal BMI is associated with the in utero growth of male offspring but not female offspring, supports this view (Chen et al., 2012; Pomeroy et al., 2015). It is also possible that mitochondrial inheritance (via the mother) (Giles et al., 1980) may play a role and elevated mother–child risks might seem to suggest this option, but the available evidence for obesity is not consistent to date (Barrett, 2001; Knoll et al., 2014; Yang et al., 2007).

Adding these two contrasting effects together, we hypothesize that mothers and fathers might generate different effects on sons compared to daughters. In this article, we investigate the intergenerational transmission of obesity by examining the association between parental BMI and their offspring's body composition. Body composition was assessed using the four‐component (4C) model, considered the most accurate in vivo approach (Wells et al., 2012) because assumptions regarding the composition of the LM are minimal (Fuller et al., 1992; Wells et al., 2012). We tested whether associations between parental BMI and offspring FM or LM are associated with the sex of the parent or the offspring.

## METHODS

We used a database of 533 children originally recruited to generate reference curves of body composition (Wells et al., 2012). Healthy children and young adults were recruited for the study from the general population using flyers and newspaper advertisements in London and the southeast of England from 2001. The age range was 4 to 23 years (young adults were included to cover the entire pediatric age range). There were no exclusion criteria for BMI. The sample was narrowed to 511 children aged 5–21 years, to match the age range for which body composition reference curves exist (we used the 20‐year‐old reference ranges for offspring aged 21 years). The methods are described elsewhere in detail (Wells et al., 2012). Briefly, all individuals attended Great Ormond Street Hospital in London for body‐composition investigation. Weight and height were measured by using standard protocols, 60% of the measurements were by one operator and the rest by five other operators. FM and LM were calculated using the 4C model which utilizes body volume (BV) by air‐displacement plethysmography (Bodpod; Cosmed, Italy), bone mineral content from dual‐energy X‐ray absorptiometry (DXA; GE Medical Systems, UK) and total body water (TBW) by deuterium dilution. The technical error of measurement (TEM) for height was 0.38 cm and TEM% was 0.23% (Ulijaszek and Kerr, 1999). Bone mineral content (BMC) in Equations (1) and (2) were used to calculate FM and LM.

(1) FM=2.747×BV−0.710×TBW+1.460×BMC−2.050×Weight

(2) LM=Weight−FM

Current parental height and weight were obtained by recall. The attending parent, usually the mother, was asked for her height and weight, and that of the other parent.

Ethical approval for the original study was granted by the Research Ethics Committee of University College London Institute of Child Health and Great Ormond Street Hospital.

### Analysis

BMI was estimated as weight (in kg) divided by squared height (in meters). LM index (LMI) and FM index (FMI) were calculated based on the relationship described in Eqs. (3) and (4).

(3) $$\frac{Weight}{{Height}^{2}}\;=\;\frac{FM}{{Height}^{2}}+\frac{LM}{{Height}^{2}}$$

(4) BMI = FMI+LMI

Exposure variables were parental BMI and outcome variables were the offspring anthropometry *z*‐scores. Offspring BMI, LMI, and FMI were converted to age and sex‐specific *z*‐scores based on the British 1990 growth references (BMI) and the new UK body composition reference child (LMI and FMI) using the lmsGrowth Excel add‐in (version 2.74) (Cole et al., 1995; Wells et al., 2012).

Associations were initially examined using correlations and then univariable and multivariable linear regression. First, the association of maternal and paternal BMI was compared with each of the offspring outcome variables for all offspring together, and then stratified by sex. The multivariable model included the other parent's BMI to account for assortative mating, where men and women do not mate at random as has been shown for fat and lean mass (Speakman et al., 2007). We calculated the variance inflation factor for these models using the function vif from R library car (Fox and Weisberg, 2011).

The data were initially limited to those for which there were complete data for both parents and offspring. Missing data points comprised approximately 14% of the paternal data, therefore we decided to impute values for the parent's BMI, using multiple imputation with chained equations (MICE) to improve our inferences on the associations between the exposure and the outcome variables (van Buuren, 2012). The offspring data were complete. We checked the assumption of data missing completely at random for every model, performing *t*‐tests on the response variable in each regression model against missing/not missing for each explanatory variable in the model. We considered 10 multiple imputations for each model.

Additional sensitivity analyses were then done to take into account potential non‐paternity using Steer's correction to Clemons’ formula: (Clemons, 2000; Steer, 2009) Let $\sigma_{mm},{\text{and}\;\sigma }_{ff}$ be the variances of the maternal and reported paternal BMI, and $\sigma_{fm}\;and\;\sigma_{bm}$ be the covariances between the maternal and reported paternal BMI, and the maternal and biological paternal BMI. Let p be the probability that the reported father is not the biological father, then a is an indicator function with value (1−p) if $\sigma_{bm}=0$, and $1\;\text{if}\;\sigma_{bm}=\sigma_{fm}$. The attenuation matrix can be written as:

(5) $$\Lambda ={\left(\begin{array}{cc} \sigma_{ff} & a\;\sigma_{ff}\\ a\;\sigma_{ff} & \sigma_{ff} \end{array}\right)}^{-1}\left(\begin{array}{cc} {\left(1-p\right)\sigma }_{ff} & {\left(1-p\right)\sigma }_{fm}\\ \sigma_{ff} & \sigma_{mm} \end{array}\right)$$

If the maternal and paternal regression coefficients are $\beta_{1}\;\text{and}\;\beta_{2}$ then the coefficients adjusted for potential non‐paternity are:

(6) $$\Lambda \left(\begin{array}{c} \beta_{1}\\ \beta_{2} \end{array}\right)=\left(\begin{array}{c} \beta_{1}^{*}\\ \beta_{2}^{*} \end{array}\right)=\left(\begin{array}{c} \frac{\left(-1+a+p\right)\beta_{2}\sigma_{fm}\sigma_{mm}+\beta_{1}\left(a\left(-1+p\right)\sigma_{fm}^{2}+\sigma_{ff}{\;\sigma }_{mm}\right)}{\left(-1+p\right)\left(\sigma_{fm}^{2}-{\sigma_{ff}{\;\sigma }_{mm}}_{\;}\right)}\\ -\frac{\left(1+a\left(-1+p\right)\right)\beta_{1}\sigma_{ff}\sigma_{fm}+\beta_{2}\left(a\sigma_{fm}^{2}+\left(-1+p\right)\sigma_{ff}{\;\sigma }_{mm}\right)}{\left(-1+p\right)\left(\sigma_{fm}^{2}-{\sigma_{ff}{\;\sigma }_{mm}}_{\;}\right)} \end{array}\right)$$

We used a = 1 as, in the absence of any further data, we considered that assuming equality of correlation (reflected in the covariance values) for the association patterns present (i) between maternal BMI and biological father's BMI and (ii) between maternal BMI and reported father's BMI was more informative than assuming lack of correlation between maternal BMI and biological father's BMI. The variances of the parental heights were calculated applying Rubin's rule to the imputed datasets, (Rubin, 1987) which gives a weighted average of the estimates obtained by fitting the regression models to each of the 10 imputed datasets.

We tested the hypothesis of no difference between maternal and paternal regression coefficients on offspring BMI, FMI, and LMI z‐scores for different assumed percentages of non‐paternity using a bootstrap procedure which took into account the MICE models. The one‐sided *p*‐values correspond to the alternative hypothesis of maternal effects being larger than paternal effects, and were obtained analyzing 10,000 realizations of the empirical resampling distribution of these differences.

Statistical analyses were carried out using Stata software version 12.1 (Stata Corporation, Texas 2011) and in the R language and environment for statistical computing version 3.0.1 (Team, 2011). Regression models accounting for missing values were fitted in R using the package mice (van Buuren and Groothuis‐Oudshoorn, 2011) to perform MICE.

## RESULTS

A description of the sample is given in Table 1. Ninety‐one percent of the offspring reported to be ethnically European, the remainder were of varied ethnic origin, including mixed ethnic origin. The mean height and weight *z*‐scores were greater than zero, with a range of >5 z‐scores. Girls were a little taller than boys but also presented more variability in their height (*t* test for difference between girls and boys: weight‐for‐age *p* = 0.18, height‐for‐age *p* = 0.09). The Pearson correlation coefficient between maternal and paternal BMI was 0.143 (*p* = 0.003). The variance inflation factor was 1.021, which did not suggest concerns regarding collinearity.

**Table 1:** Participant characteristics

Variable	Boys		Girls	
Number	248		263	
Ethnicity	n (%)		*n* (%)	
European	221 (89.1%)		242 (92.0%)	
Asian	8 (3.2%)		2 (0.8%)	
Black	10 (4.0%)		12 (4.6%)	
Chinese	3 (1.2%)		4 (1.5%)	
Other	6 (2.4%)		3 (1.1%)	
	Mean (SD)	Range	Mean (SD)	Range
Age (years)	12.3 (4.0)	5.1 to 21.8	12.8 (4.3)	5.0 to 21.9
Weight (kg)	45.2 (19.3)	16.1 to 106.4	46.1 (16.4)	17.0 to 90.8
Weight *z*‐score	0.29 (1.08)	−2.42 to 3.44	0.42 (1.10)	−2.75 to 3.46
Height (cm)	150.7 (20.5)	104.7 to 190.4	149.9 (17.8)	107.0 to 181.7
Height *z*‐score	0.20 (0.96)	−2.09 to 3.28	0.35 (1.03)	−2.77 to 3.42
BMI (kg/m^2^)	18.9 (3.9)	13.0 to 37.9	19.8 (3.8)	12.8 to 34.5
BMI *z*‐score	0.22 (1.16)	−2.99 to 3.49	0.31 (1.14)	−3.33 to 3.09
Fat mass (kg)	9.1 (6.8)	1.0 to 45.5	13.1 (7.6)	2.3 to 40.0
FMI *z*‐score	0.01 (0.99)	−3.28 to 2.34	0.00 (1.00)	−2.41 to 2.41
Lean mass (kg)	36.1 (15.0)	13.7 to 76.4	33.0 (10.2)	13.2 to 61.9
LMI *z*‐score	−0.01 (1.08)	−4.3 to 5.13	0.01 (1.10)	−3.23 to 2.77
Mother's BMI (kg/m^2^) (Range = Minimum, 1st to 4th quintiles, maximum)	25.5 (4.7)	17.0, 21.7, 23.3, 25.2, 29.5, 41.9	25.3 (5.2)	16.0, 21.6, 23.2, 25.0, 28.5, 43.9
Father's BMI (kg/m^2^) (Range = Minimum, 1st to 4th quintiles, maximum)	26.5 (3.7)	16.9, 23.5, 25.1, 27.3, 29.7, 37.3	26.3 (4.1)	17.7, 22.8, 24.7, 26.7, 29.3, 37.4

**Figure 1:**
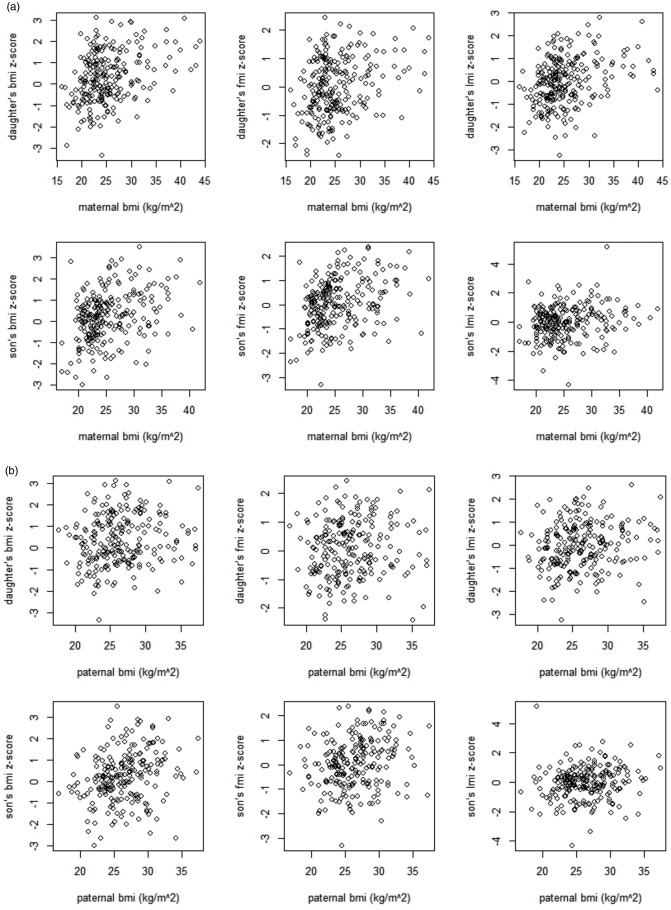
Scatterplots to show the maternal (1a) and paternal BMI (1b) and offspring body composition.

The data are shown in scatterplots for each parent separately in Figures 1a,b. When considering sons and daughters together, there was a positive association between both parent's BMI and offspring BMI, FMI and LMI (Table 2). This association was present in both the univariable and adjusted model (Table 2 and Supporting Information Table 1a) and was present with and without imputation (Supporting Information Table 1b). The regression coefficients for BMI, LMI, and FMI for maternal BMI tended to be approximately one and a half to two times that of the paternal BMI. The associations of maternal and paternal BMI were similar for both FMI and LMI of the offspring.

**Table 2:** Sex‐specific associations of offspring body composition (BMI, FMI, LMI) with parental BMI

Offspring body composition		Predicted change in offspring body composition, β (95% CI), associated with parental BMI^a^			
		Mother's BMI		Father's BMI	
		Unadjusted	Adjusted^b^	Unadjusted	Adjusted^c^
All offspring	BMI *z*‐score	0.078 (0.059 to 0.097)	0.073 (0.053 to 0.093)	0.052 (0.024 to 0.080)	0.038 (0.011 to 0.065)
	FMI *z*‐score	0.061 (0.044 to 0.079)	0.056 (0.039 to 0.074)	0.031 (0.006 to 0.056)	0.029 (0.007 to 0.051)
	LMI *z*‐score	0.053 (0.034 to 0.071)	0.049 (0.030 to 0.067)	0.036 (0.012 to 0.061)	0.029 (0.003 to 0.055)
Female offspring	BMI *z*‐score	0.075 (0.049 to 0.100)	0.071 (0.044 to 0.097)	0.032 (−0.004 to 0.069)	0.029 (−0.005 to 0.063)
	FMI *z*‐score	0.057 (0.033 to 0.080)	0.055 (0.031 to 0.078)	0.019 (−0.020 to 0.058)	0.017 (−0.017 to 0.051)
	LMI *z*‐score	0.060 (0.037 to 0.083)	0.057 (0.033 to 0.081)	0.037 (0.004 to 0.070)	0.028 (−0.004 to 0.061)
Male offspring	BMI *z*‐score	0.081 (0.051 to 0.110)	0.074 (0.045 to 0.104)	0.072 (0.031 to 0.112)	0.057 (0.018 to 0.096)
	FMI *z*‐score	0.068 (0.043 to 0.094)	0.064 (0.038 to 0.090)	0.054 (0.019 to 0.090)	0.041 (0.006 to 0.077)
	LMI *z*‐score	0.043 (0.013 to 0.072)	0.040 (0.010 to 0.070)	0.040 (0.001 to 0.078)	0.029 (−0.011 to 0.070)

a Results with multiple imputation, per one‐unit increase.

b Adjusted for father's BMI.

c Adjusted for mother's BMI.

**Figure 2:**
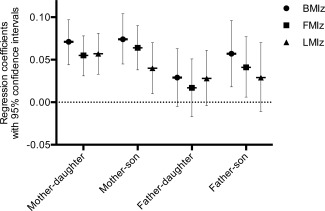
Associations of childhood *z*‐scores for BMI, FMI, and LMI with Parental BMI: regression coefficients with 95% confidence intervals (with imputation).

When considering boys and girls separately, the association of the mother's BMI with the BMI of both boys and girls was statistically significant in all cases and of a similar magnitude for both. While not showing definitive evidence for this, there was a suggestion of a greater association with FMI than LMI in boys. In girls there was a marginally greater association with LMI. For the father's BMI there was only a significant association with the BMI, FMI and LMI *z*‐scores of boys. The magnitude of the association for boys was similar to that of the mother's association. Again there was a suggestion of a greater association with FMI in boys and LMI in girls and this difference was maintained with imputed results. Of interest, significant associations were not seen between the father's BMI and any of the daughter's tested indices. The associations for each parent and child are shown in Figure 2.

Models fitted with multiple imputation showed a slight strengthening of the association of the mother's BMI and weakening of the father's BMI for all offspring outcome variables. It seemed a fair assumption that the missing data occurred completely at random. The *p‐*values testing for a difference between the mean values of missing and not missing data showed an inability to reject the null hypothesis on most occasions, as shown in Supporting Information Table 2. With a *p‐*value of 0.03, it is possible that the association between father's BMI and daughter's FMI did not achieve this criteria. We are aware, however, that it is not possible to directly test this assumption and that this seemingly significant result may have occurred by chance. We have therefore continued to use this imputed data, but treat this particular result with caution. The main conclusion from this approach was the same as in the non‐imputed one.

The results for different levels of non‐paternity are shown in Supporting Information Table 3. In each case, the maternal regression coefficient falls and the paternal rises with increasing levels of non‐paternity. Maternal coefficients remained greater than paternal throughout, but the *p‐*values indicated no statistical difference at greater levels of non‐paternity.

## DISCUSSION

Our analyses produced two important findings. First, overall we found a stronger association of maternal than paternal BMI with offspring body composition, though the strength of this effect varied across outcomes and was statistically significant in daughters only. Second, we found that the association between paternal phenotype and that of the offspring differed between sons and daughters, being significant in the former but not the latter. Therefore, both hypotheses are supported, in that the two parents generate different associations, and sons and daughters are also associated with their parents in different ways. Previous findings on these issues have been inconsistent, despite several studies on a very large sample size, hence we briefly review these findings.

### BMI

A review of the literature by Patro et al. (2013) looked at the effect of prenatal maternal versus paternal BMI on offspring BMI or adiposity over the age of 5 years. They tended to find a greater maternal association with offspring BMI but showed inconsistent findings and daughters showed equal or different associations (Patro et al., 2013). Our findings are in keeping with studies by Lawlor et al. (2007), Whitaker et al. (2010), Danielzik et al. (2002), Linabery et al. (2013), and Svensson et al. (2011; at seven years of age) who showed a greater association with the mother's BMI than that of the father's. As described previously, this may be explained by biological or behavioral mechanisms, as mothers tend to be more involved in all aspects of child rearing.

We also showed the association of the father's BMI with sons was a little lower, but similar to that of the mother's, whereas there was no association between the father's BMI and that of the daughters. Regarding the father/mother to daughter/son relationship, our results mirror Power et al. (2011) who found a lower correlation coefficient for father‐to‐daughter than mother‐to‐daughter or ‐son or father‐to‐son. The results however differ from Burke et al. (2001) who suggested a greater association of father‐to‐daughter. Jääskeläinen et al. (2011) similarly showed a greater father‐daughter association for parental BMI measured prior to the child's conception, but not when estimated at the same time as offspring measurement. The other studies in Table 3 tend to show no sex‐specific difference or stronger associations between one parent and offspring (except Perez‐Pastor et al. (2009) who show a stronger association between mother‐to‐daughter and father‐to‐son).

**Table 3:** Summary of the evidence on the effect of parental BMI on offspring BMI

First author and journal	Type of study	Number of participants & location	Exposure variable	Outcome	Results
Linabery et al., Pediatr Obesity 2013	Cohort	912 parent and child trios, USA	Parental BMI by measurement close to birth	Repeated child BMI measurements	Maternal obesity had a greater association with offspring BMI than paternal obesity in early life.
Fleten et al., Am J Epi 2012	Cohort	29,216 parent and child trios, Norway	Pre‐pregnancy BMI from self‐reported parental height and weight	Mother's report of the child's height and weight at age 3	No difference between maternal or paternal BMI and offspring's BMI. A one standard deviation increase in maternal and paternal BMI led to a 0.12 and 0.13 increase in the offspring's BMI, respectively.
Morandi et al., Plos One 2012	Cohort	4,032 parent and child trios, Finland	Pre‐pregnancy parental BMI. Not stated how this was collected.	Offspring BMI from measured height and weight at age 7 and 16.	Controlling for other predictors in multiple logistic regression, maternal BMI to childhood overweight‐obesity OR = 1.13 (95% CI 1.10, 1.16) and adolescent overweight–obesity OR = 1.17 (95% CI 1.14,1.20). Paternal BMI to childhood overweight–obesity OR = 1.11 (95% CI 1.08, 1.15) and adolescent overweight–obesity OR = 1.12 (95% CI 1.09,1.15).
Jääskeläinen et al., Int J Obes 2011	Cohort	4,788 parent and child trios, Finland	BMI from self‐reported parental height and weight obtained prenatally and at the same time as the child's	Offspring BMI from measured height and weight at age 16.	Cohort analyses: Parental pre‐pregnancy obesity and offspring overweight: mother–son OR 4.36 (95% CI 2.50,7.59), mother–daughter OR 3.95 (95% CI 2.34,6.68), father–son OR 3.17 (CI 1.70,5.92); father–daughter: OR 5.58 (95% CI 3.09,10.07). Cross‐sectional analyses: Parental obesity and offspring overweight: mother–son OR 4.60 (95% CI 3.24,6.53), mother–daughter OR 5.04 (95% CI 3.56,7.14), father–son OR 3.05 (CI 2.08,4.49); father–daughter: OR 3.72 (95% CI 2.56,5.59).
Patel et al., Plos One 2011	Cross‐sectional	12,181 parent and child trios, Belarus	BMI from self‐reported parental height and weight at the same time as the child's measurement	Offspring BMI from measured height and weight at age 6.5.	No difference in association between mother and daughter or son or between father and daughter or son.
Svensson et al., Int J Obesity 2011	Cohort	3,340 parent and child trios, selected from children who attended the National Childhood Obesity Centre, Sweden	BMI from self‐reported parental height and weight at the same time as the child's measurement	Offspring BMI from measured height and weight at age 7 and 15.	The severity of obesity at age 7 years, adjusted for several relevant factors, related significantly with the maternal BMI, but not with paternal BMI. Severity of obesity was significantly correlated with both maternal and paternal BMI at age 15 years. No difference between parent and daughter/son association.
Power et al., Ann Human Biol 2011	Cohort	17,000 from the original cohort. 4,271 individuals were later selected who had children, UK	BMI from self‐reported parental height and weight at offspring age 11.	Offspring BMI from measured height and weight at age 7, 11, 16, and 33.	The correlation coefficent is similar for mother to daughter (0.23), mother to son (0.21) and father to son (0.21), but was lower for father to daughter (0.14).
Whitaker et al., Am J Clin Nut 2010	Cross‐sectional	4,423 parent and child trios, UK	BMI from measured height and weight in both parents at the same time as offspring	Offspring BMI from measured height and weight at age 2–15 years	Mother to offspring association is stronger than father to offspring, but no difference for daughters or sons. Partial correlations (*r*) were controlled for the other parent's BMI. Mother to daughter = 0.26; mother to son = 0.22; father to daughter = 0.19; father to son = 0.19.
Leary et al., Int J Obesity 2010	Cohort	4,654 parent and child trios, UK	BMI from self‐reported parental height and weight before birth	Offspring BMI from measured height and weight at age 7.5. BMI values were age adjusted for all values using the residuals from gender specific linear regressions of BMI on age.	Mother to daughter association (β = 0.18 (95% CI = 0.16–0.20) is a little stronger than mother to son (β = 0.13 (95% CI = 0.12–0.15), but the father has a similar association with both children.
Perez‐Pastor et al., Int J Obesity 2009	Cohort	226 parent and child trios, UK	BMI from measured height and weight in both parents when offspring was aged 5	Offspring BMI from measured height and weight at age 5–8 years.	Mother to daughter association (1.37 SD scores for daughters v 0.16 SD scores for sons) and father to son (0.17 SD scores for daughters vs. 1.28 SD scores for sons).
Kivimaki et al., Am J Clin Nutr 2007	Cohort	2,980 complete and child trios, Finland	BMI from self‐reported parental height at the same time as offspring	Offspring BMI from measured height and weight at age 3–18 years.	There is a similar association of maternal or paternal BMI and son or daughter BMI.
Lawlor et al., Am J Epidemiol 2007	Cohort	3,340 parent and child trios, Australia	BMI from self‐reported parental height and weight before birth	Offspring BMI from measured height and weight at age 14 years	Mother to child association is stronger than father to child. For a one standard deviation increase in the child's BMI, there was a 0.362 SD increase in maternal BMI (95% CI: 0.323–0.402) and a 0.239 SD increase in paternal BMI (95% CI: 0.197–0.282).
Danielzik et al., Eur J Nutr 2002	Cross‐sectional	3,306 parent and child trios, Germany	BMI from self‐reported parental height and weight at the same time as offspring	Offspring BMI from measured height and weight at age 5–7 years	Stronger correlation between maternal than paternal BMI with the child's BMI. The correlation is similar for daughters and sons. Mother to daughter *r* = 0.242; mother to son *r* = 0.254; father to daughter *r* = 0.159; father to son *r* = 0.160.

Overall, no consistent pattern has emerged from research on this issue. Given the multiple different routes through which each parent can affect the phenotype of their offspring (Wells, 2014), it is very plausible that the association between parental phenotype and offspring BMI is genuinely heterogeneous across populations, as has been shown previously for the association between infant weight gain and later body composition (Wells et al., 2007).

### Fat and lean mass

Our research goes further than most previous work in that it investigates whether an association exists between parental BMI and FM and LM in the offspring, using the 4C model. We found that the mother's BMI shows similar associations with that of her sons’ and daughters’ body composition. For fathers, no statistically significant associations were found with girls, in contrast to boys, where an association was seen with FM. Interestingly, among daughters there were similar associations for FM and LM, whereas in boys, with both parents, there was a greater association with FM than LM.

We found two studies that investigated associations between parental BMI and offspring FM and LM. Lawlor et al. (2008) assessed children at ages 9–11 years using DXA scans. They also found a strong association between parental BMI and offspring adiposity that was greater for mothers than fathers, and a weaker association between parental BMI and child LM that was of similar magnitude for each parent (Lawlor et al., 2008). In a cohort study from India, Veena et al. (2012) generally showed no difference between maternal adiposity during pregnancy or paternal adiposity when children were 5 years old and the adiposity of daughters or sons measured by bioelectrical impedance at age 9 years.

From an evolutionary perspective, differential effects of the two parents on the body composition of their offspring are expected to derive from fathers and mothers using different strategies to maximize their reproductive fitness. While the mother invests nutritional resources directly during early life, the father only invests information that may manipulate maternal physiology in his interests (Haig, 1993). Sex‐different responses of the offspring are further predicted, because lean mass and fat mass bring different reproductive pay‐offs for sons versus daughters (Wells et al., 2011).

### Strengths

The main strength of our study was the use of the gold‐standard 4C model to produce estimates of FM and LM in children. This allows us to predict more accurately the associations of parental BMI with different tissues. We have also accounted for assortative mating using a multiple regression model, and compensated for missing data by using multiple imputation. We have then tested our assumptions by giving estimates of the effects at different levels of non‐paternity.

### Limitations

The main limitation was the estimation of parental BMI by recall. Parental recall is likely to have resulted in error in the parental height and weight estimates, but a potential bias may work in either direction and would not explain the difference in results for sons and daughters, in that we would not anticipate recall error to be associated with offspring sex. Additionally, our findings are similar to some other studies that have measured parental anthropometry (Table 3). As described previously, several mechanisms exist by which environmental or genetic processes could allow parental phenotype to influence different body tissue types in sons and daughters. Further research is needed to elucidate in more detail the heterogeneity of findings on this research issue. We were unable to consider whether the association between the parent's BMI and offspring body composition varied within an individual over time. It is plausible that this association is different in early life than later in childhood and this is an important area of future research.

## SUMMARY

Our work indicates a stronger association of maternal than paternal BMI with offspring body composition, and the suggestion that paternal associations may differ between sons and daughters. Given that parental effects on offspring BMI are established to be a strong predictor of childhood obesity (Parsons et al., 1999), our findings indicate that multiple and complex mechanisms contribute. Further work on this issue is required in order to improve understanding of how obesity prevention strategies may be developed to minimize the intergenerational transmission of childhood obesity.

## Supporting information

Supporting Information
