# Supplementary material for: Disentangling the associations between parental BMI and offspring body composition using the four‐component model
Source: Am J Hum Biol. 2016 Feb 5;28(4):524–33. doi: 10.1002/ajhb.22825 (PMC4957621; doi:10.1002/ajhb.22825)
Supplement: Supplementary file 1 — Supporting Information [file AJHB-28-524-s001.docx]

# The association of parental BMI and offspring body composition using the four-component model

Devakumar D, Grijalva-Eternod C, Cortina-Borja M, Willams JE, Fewtrell MS, Wells JCK

**Webtable 1: Univariable and multivariable regression coefficients for the association between maternal or paternal BMI and offspring body composition, with (a) no imputation and (b) with multiple imputation**

|  |  | Univariable linear regression | | Multivariable linear regression adjusted for the other parent’s BMI | |
| --- | --- | --- | --- | --- | --- |
| (a) |  | Mother’s BMI (95% CI) | Father’s BMI (95% CI) | Mother’s BMI (95% CI) | Father’s BMI (95% CI) |
| Offspring together | BMI z-score | 0.071 (0.050 to 0.093) | 0.053 (0.025 to 0.081) | 0.067 (0.046 to 0.088) | 0.041 (0.014 to 0.068) |
|  | FMI z-score | 0.054 (0.036 to 0.073) | 0.036 (0.012 to 0.059) | 0.052 (0.033 to 0.070) | 0.026 (0.003 to 0.049) |
|  | LMI z-score | 0.049 (0.029 to 0.069) | 0.036 (0.010 to 0.062) | 0.046 (0.026 to 0.066) | 0.028 (0.002 to 0.053) |
| Daughters | BMI z-score | 0.069 (0.040 to 0.097) | 0.037 (-0.001 to 0.074) | 0.066 (0.037 to 0.094) | 0.024 (-0.012 to 0.061) |
|  | FMI z-score | 0.047 (0.023 to 0.072) | 0.019 (-0.013 to 0.051) | 0.046 (0.021 to 0.071) | 0.011 (-0.021 to 0.042) |
|  | LMI z-score | 0.058 (0.032 to 0.083) | 0.028 (-0.003 to 0.060) | 0.054 (0.029 to 0.080) | 0.025 (-0.007 to 0.058) |
| Sons | BMI z-score | 0.075 (0.043 to 0.107) | 0.074 (0.032 to 0.116) | 0.069 (0.037 to 0.100) | 0.062 (0.021 to 0.102) |
|  | FMI z-score | 0.063 (0.036 to 0.090) | 0.056 (0.021 to 0.092) | 0.058 (0.032 to 0.085) | 0.046 (0.011 to 0.080) |
|  | LMI z-score | 0.039 (0.008 to 0.071) | 0.036 (0.003 to 0.069) | 0.036 (0.004 to 0.067) | 0.031 (-0.010 to 0.072) |
| (b) |  |  |  |  |  |
| Offspring together | BMI z-score | 0.078 (0.059 to 0.097) | 0.052 (0.024 to 0.080) | 0.073 (0.053 to 0.093) | 0.038 (0.011 to 0.065) |
|  | FMI z-score | 0.061 (0.044 to 0.079) | 0.031 (0.006 to 0.056) | 0.056 (0.039 to 0.074) | 0.029 (0.007 to 0.051) |
|  | LMI z-score | 0.053 (0.034 to 0.071) | 0.036 (0.012 to 0.061) | 0.049 (0.030 to 0.067) | 0.029 (0.003 to 0.055) |
| Daughters | BMI z-score | 0.075 (0.049 to 0.100) | 0.032 (-0.004 to 0.069) | 0.071 (0.044 to 0.097) | 0.029 (-0.005 to 0.063) |
|  | FMI z-score | 0.057 (0.033 to 0.080) | 0.019 (-0.020 to 0.058) | 0.055 (0.031 to 0.078) | 0.017 (-0.017 to 0.051) |
|  | LMI z-score | 0.060 (0.037 to 0.083) | 0.037 (0.004 to 0.070) | 0.057 (0.033 to 0.081) | 0.028 (-0.004 to 0.061) |
| Sons | BMI z-score | 0.081 (0.051 to 0.110) | 0.072 (0.031 to 0.112) | 0.074 (0.045 to 0.104) | 0.057 (0.018 to 0.096) |
|  | FMI z-score | 0.068 (0.043 to 0.094) | 0.054 (0.019 to 0.090) | 0.064 (0.038 to 0.090) | 0.041 (0.006 to 0.077) |
|  | LMI z-score | 0.043 (0.013 to 0.072) | 0.040 (0.001 to 0.078) | 0.040 (0.010 to 0.070) | 0.029 (-0.011 to 0.070) |

**Webtable 2: Mean z score for complete (n=439) and missing data (n=72) and *p* values for the null hypothesis of no difference between them**

|  | | Univariable linear regression | | | | | | Multivariable linear regression* | | | | | |
| --- | --- | --- | --- | --- | --- | --- | --- | --- | --- | --- | --- | --- | --- |
|  |  | Mother’s BMI | | | Father’s BMI | | | Mother’s BMI | | | Father’s BMI | | |
|  |  | Complete data mean | Missing mean | *p* value | Complete data mean | Missing mean | *p* value | Complete data mean | Missing mean | P value | Complete data mean | Missing mean | *p* value |
| Offspring together | BMI z score | 0.028 | 0.026 | 0.960 | 0.293 | 0.108 | 0.169 | 0.268 | 0.260 | 0.960 | 0.293 | 0.108 | 0.169 |
|  | FMI z score | 0.009 | -0.102 | 0.559 | 0.047 | -0.271 | 0.013 | 0.009 | -0.102 | 0.559 | 0.047 | -0.271 | 0.013 |
|  | LMI z score | -0.010 | 0.158 | 0.311 | -0.018 | 0.118 | 0.294 | -0.010 | 0.158 | 0.311 | -0.018 | 0.118 | 0.294 |
| Daughters | BMI z score | 0.304 | 0.354 | 0.801 | 0.352 | 0.065 | 0.124 | 0.304 | 0.354 | 0.801 | 0.352 | 0.065 | 0.124 |
|  | FMI z score | 0.005 | -0.094 | 0.691 | 0.057 | -0.334 | 0.034 | 0.005 | -0.094 | 0.691 | 0.057 | -0.334 | 0.034 |
|  | LMI z score | -0.015 | 0.287 | 0.125 | -0.002 | 0.0715 | 0.667 | -0.015 | 0.287 | 0.125 | -0.002 | 0.072 | 0.667 |
| Sons | BMI z score | 0.230 | 0.095 | 0.641 | 0.233 | 0.162 | 0.720 | 0.230 | 0.095 | 0.641 | 0.233 | 0.162 | 0.720 |
|  | FMI z score | 0.014 | -0.116 | 0.677 | 0.037 | -0.192 | 0.193 | 0.014 | -0.116 | 0.677 | 0.037 | -0.192 | 0.1931 |
|  | LMI z score | -0.005 | -0.067 | 0.840 | -0.035 | 0.176 | 0.297 | -0.005 | -0.067 | 0.840 | -0.035 | 0.176 | 0.297 |

*Multi-variable model is adjusted for the other parent’s indices.

Webtable 3: The association between maternal and paternal BMI and offspring BMI, FMI and LMI z-scores regression coefficients at increasing percentages of non-paternity

|  |  | **Level of non-paternity** | **Multivariable linear regression adjusted for the other parent’s BMI *** | | ***p* value**** |
| --- | --- | --- | --- | --- | --- |
|  |  |  | **Maternal coefficient** | **Paternal coefficient** |  |
| **Offspring together** | BMI z-score | 0% | 0.073 | 0.038 | 0.035 |
|  |  | 5% | 0.073 | 0.041 | 0.055 |
|  |  | 10% | 0.073 | 0.044 | 0.085 |
|  |  | 15% | 0.072 | 0.047 | 0.131 |
|  | FMI z-score | 0% | 0.056 | 0.029 | 0.023 |
|  |  | 5% | 0.056 | 0.031 | 0.032 |
|  |  | 10% | 0.056 | 0.033 | 0.049 |
|  |  | 15% | 0.056 | 0.036 | 0.073 |
|  | LMI z-score | 0% | 0.049 | 0.029 | 0.114 |
|  |  | 5% | 0.048 | 0.031 | 0.145 |
|  |  | 10% | 0.048 | 0.033 | 0.183 |
|  |  | 15% | 0.048 | 0.036 | 0.228 |
| **Daughters** | BMI z-score | 0% | 0.071 | 0.029 | 0.016 |
|  |  | 5% | 0.071 | 0.032 | 0.023 |
|  |  | 10% | 0.070 | 0.034 | 0.035 |
|  |  | 15% | 0.070 | 0.037 | 0.052 |
|  | FMI z-score | 0% | 0.055 | 0.017 | 0.024 |
|  |  | 5% | 0.054 | 0.018 | 0.033 |
|  |  | 10% | 0.054 | 0.020 | 0.042 |
|  |  | 15% | 0.054 | 0.022 | 0.058 |
|  | LMI z-score | 0% | 0.057 | 0.028 | 0.058 |
|  |  | 5% | 0.057 | 0.030 | 0.077 |
|  |  | 10% | 0.057 | 0.033 | 0.103 |
|  |  | 15% | 0.056 | 0.035 | 0.133 |
| **Sons** | BMI z-score | 0% | 0.074 | 0.057 | 0.335 |
|  |  | 5% | 0.074 | 0.060 | 0.395 |
|  |  | 10% | 0.074 | 0.065 | 0.458 |
|  |  | 15% | 0.073 | 0.069 | 0.522 |
|  | FMI z-score | 0% | 0.064 | 0.041 | 0.189 |
|  |  | 5% | 0.064 | 0.044 | 0.234 |
|  |  | 10% | 0.064 | 0.047 | 0.281 |
|  |  | 15% | 0.063 | 0.051 | 0.335 |
|  | LMI z-score | 0% | 0.040 | 0.029 | 0.406 |
|  |  | 5% | 0.040 | 0.031 | 0.431 |
|  |  | 10% | 0.040 | 0.034 | 0.462 |
|  |  | 15% | 0.039 | 0.036 | 0.492 |

* Coefficients refer to the association between parental BMI and offspring body composition in scenarios of assumed non-paternity.

** One-sided p-values corresponding to the alternative hypothesis of maternal effects being larger than paternal effects.
